# Preparation and Anti-Tumour Activity of Some Arylbismuth(III) Oxine Complexes

**DOI:** 10.1155/MBD.1998.295

**Published:** 1998

**Authors:** Katharine A. Smith, Glen B. Deacon, W. Roy Jackson, Edward R. T. Tiekink, Silvina Rainone, Lorraine K. Webster

**Affiliations:** 1 Department of Chemistry Monash University Clayton Victoria 3168 Australia; 2 Department of Chemistry The University of Adelaide 5005 Australia; 3 Research Division Peter MacCallum Cancer Institute Melbourne Victoria 3002 Australia

## Abstract

New arylbismuth(lll) oxinates, PhBi(MeOx)_2_, (p-MeC_6_H_4_)Bi(Ox)_2_, (p-MeC_6_H_4_)Bi(MeOx)_2_, (p-ClC_6_H_4_)Bi(Ox)_2_, and (p-ClC_6_H_4_)Bi(MeOx)_2_ (Ox^−^ = quinolin-8-olate and MeOx^−^=2-methylquinolin-8-olate) have been prepared by reaction of the appropriate diarylbismuth chlorides with Na(Ox) or Na(MeOx) in the presence of 15-crown-5. An X-ray crystallographic study has shown PhBi(MeOx)_2_ to be a five coordinate monomer with distorted square pyramidal stereochemistry. Chelating MeOx ligands have a cisoid arrangement in the square plane and the phenyl group is apical. The lattice is stabilised by significant π-π interactions between centrosymmetric molecules. A range of these complexes has been shown to have high *in vitro* biological activity (comparable with or better than cisplatin) against L1210 leukaemia, the corresponding cisplatin resistant line, and a human ovarian cell line, SKOV-3. However, initial *in vivo* testing against a solid mouse plasmacytoma (PC6) and P388 leukaemia has not revealed significant activity.


### PREPARATION AND ANTI-TUMOUR ACTIVITY OF SOME ARYLBISMUTH(III) OXINE COMPLEXES

Katharine A. Smith[1], Glen B. Deacon[1], W. Roy Jackson[1], Edward R.T. Tiekink[2], Silvina Rainone[3] and Lorraine K. Webster[3]

[1]Department of Chemistry, Monash University, Clayton, Victoria, Australia 3168
[2]Department of Chemistry, The University of Adelaide, Australia 5005
3 Research Division, Peter MacCallum Cancer Institute, Melbourne, Victoria, Australia 3002

### Abstract

New arylbismuth(III) oxinates, $PhBi(MeOx)_2$, $(p\text{-}MeC_6H_4)Bi(Ox)_2$, $(p\text{-}MeC_6H_4)Bi(MeOx)_2$, $(p\text{-}ClC_6H_4)Bi(Ox)_2$, and $(p\text{-}ClC_6H_4)Bi(MeOx)_2$ ($Ox^-$ = quinolin-8-olate and $MeOx^-$ = 2-methylquinolin-8-olate) have been prepared by reaction of the appropriate diarylbismuth chlorides with Na(Ox) or Na(MeOx) in the presence of 15-crown-5. An X-ray crystallographic study has shown $PhBi(MeOx)_2$ to be a five coordinate monomer with distorted square pyramidal stereochemistry. Chelating MeOx ligands have a cisoid arrangement in the square plane and the phenyl group is apical. The lattice is stabilised by significant $\pi$-$\pi$ interactions between centrosymmetric molecules.

A range of these complexes has been shown to have high *in vitro* biological activity (comparable with or better than cisplatin) against L1210 leukaemia, the corresponding cisplatin resistant line, and a human ovarian cell line, SKOV-3. However, initial *in vivo* testing against a solid mouse plasmacytoma (PC6) and P388 leukaemia has not revealed significant activity.

### Introduction

It has been shown recently that metathetical reactions of $Ph_2BiX$ and $PhBiX_2$ (X = Cl, Br, or I) with sodium quinolin-8-olate (NaOx) are complicated by rearrangement reactions and by incomplete elimination of sodium halides [1]. Typically bimetallics of composition $PhBi(Ox)_2(NaX)_n$ were isolated together with varying amounts of $Bi(Ox)_3$. However, halide-free $PhBi(Ox)_2$ was isolated from the reaction of $Ph_2BiCl$ with Na(Ox) in the presence of 15-crown-5 and from reaction of $PhBiI_2$ with $Bu_4N(Ox)$. The low solubility of $PhBi(Ox)_2$ suggested an associated structure but no clear structural conclusions could be drawn from spectroscopic data.

We now report the synthesis of a range of pure organobismuth quinolin-8-olate and 2-methylquinolin-8-olate complexes, $RBi(Ox)_2$ and $RBi(MeOx)_2$, the X-ray crystal structure of $PhBi(MeOx)_2$ and a preliminary survey of the anti-cancer activity of these quinolin-8-olate complexes together with some related reference compounds.

There has been sporadic interest in antitumour properties of bismuth compounds [2-7] the most successful of which have been methylbismuth(III) thiolates. In addition, it has been shown that 8-quinolinol has antitumour activity [8,9]. Bismuth compounds are much more widely used in the treatment of gastrointestinal disorders [7] and of peptic ulcers [10]. The low toxicity of most bismuth compounds, especially relative to that of many other heavy metal compounds, makes them attractive candidates for drug usage [7]. It has been observed, however, that regular ingestion of high doses of bismuth salts (5 - 15 g/day by many French citizens) led to *ca* 300 cases of bismuth(III) intoxication with some deaths in 1976 [11].

### Experimental

### *Instruments and Procedures*

Details have been described previously [1].

### *Reagents*

Sodium salts of quinolin-8-ol and 2-methylquinolin-8-ol were prepared as described previously [1]. The following diarylbismuth chlorides were prepared by reaction of stoichiometric amounts of $BiCl_3$ and $Ar_3Bi$ [12]: $Ph_2BiCl$, m.p. 185-186 (dec.) lit. [13] 184-185°C;

$(4\text{-}CH_3C_6H_4)_2BiCl$, m.p. 182°C (dec.) lit. [14] 181°C; $(4\text{-}ClC_6H_4)_2BiCl$, $^1H$ nmr (300 MHz) δ, 7.57, d, J8.3 Hz, 4H, H3,5; 8.36, d, J8.2 Hz, 4H, H2,6. The compounds $Bi(Ox)_3$, $Bi(Ox)_2I$, $PhBiI_2$, $Ph_2BiI$, $PhBi(Ox)_2{\cdot}EtOH$, [M{PhBi(Ox)$_3$}] (M = Na or K) were available from an earlier study [1], and $Ph_3Bi$ was from Warman International.

### *Reactions of diarylbismuth chlorides with oxinate salts, $Ph_2BiCl$ + Na(MeOx)*

A solution of Na(MeOx) (0.98 g, 5.4 mmol) in ethanol (25 ml) with 15-crown-5 ether (2.38 g, 2.1 ml, 10.8 mmol) was added to a suspension of $Ph_2BiCl$ (2.15 g, 5.4 mmol) in ethanol (10 ml). The yellow reaction mixture was stirred for 2 h then filtered. The yellow solid was collected, washed with ethanol (3 x 5 ml), light petroleum (3 x 10 ml) and dried to yield bis(2-methylquinolin-8-olato)phenylbismuth(III) (1.45 g, 89%)† (Found: C, 51.6; H, 3.4; N, 4.6. Calc. for $C_{26}H_{21}BiN_2O_2$: C, 51.8; H, 3.5; N, 4.7%). $\nu_{max}$ 1599w, 1585m, 1557s, 1502s, 1427s, 1327s, 1294m, 1270s, 1234w, 1212w, 1186w, 1169w, 1102vs, 1055m, 1032w, 998m, 966m, 916w, 871m, 844s, 826s, 790m, 751s, 737vs, 724s, 691m, 605m $cm^{-1}$. $^1H$ nmr (300 MHz) δ 2.83, s, 6H, $CH_3$; 6.91, d, *J* 7.7 Hz, 2H, H7; 7.05, m, 3H, H4', 5; 7.44, m, 6H, H3', 5', 3, 6; 8.16, d, *J* 7.3 Hz, 2H, H2', 6'; 8.31, d, *J* 8.4 Hz, 2H, H4. $^{13}C$ nmr δ 24.14, $CH_3$; 112.95, C7; 117.31, C5; 122.52, C3; 127.38, C4'; 128.48, C6; 129.06, C4a; 131.57, C3', 5'; 134.77, C2', 6'; 138.22, C4; 141.71, C8a; 154.36, C2; 162.10, C8; C1' not observed.‡ Mass spectrum *m/z* 602 (<1%, M), 525 (6, $Bi(MeOx)_2^+$), 444 (22, $PhBi(MeOx)^+$), 367 (18, $Bi(MeOx)^+$), 286 (18, $PhBi^+$), 209 (63, $Bi^+$), 159 (100, $MeOxH^+$), 154 (44, $Ph_2^+$), 131 (63), 103 (18), 77 (30, $Ph^+$), 51 (23, $C_4H_3^+$). UV/Vis (2.07 x $10^{-4}$ M in $(CH_3)_2SO$) 320 (sh) (ε 4406), 337 (sh) (ε 5097), 371 nm (e6908) (solid state) 402 nm. The solvent was removed from the filtrate to yield a yellow solid which was extracted with water (6 x 10 ml) and ether (6 x 10 ml). NaCl and 15-crown-5 ether (2.92 g, *ca.* 100%) were isolated from the water extraction. Removal of the solvent from the ether extraction yielded $Ph_3Bi$ (1.22 g, *ca.* 100%) identified by $^1H$ n.m.r. spectroscopy.

### *$(4\text{-}CH_3C_6H_4)_2BiCl$ + Na(Ox)*

A solution of Na(Ox) (0.89 g, 5.3 mmol) with 15-crown-5 ether (2.34 g, 2.1 ml, 10.6 mmol) in ethanol (25 ml) was added to a suspension of $(4\text{-}MeC_6H_4)_2BiCl$ (2.27 g, 5.3 mmol) in ethanol (20 ml). The reaction mixture became a clear yellow colour before a yellow precipitate appeared. After stirring for 2 h, the reaction mixture was filtered and the resulting yellow solid was washed with ethanol (3 x 5 ml) and light petroleum (3 x 10 ml). This solid was further stirred for 1 h in ether (50 ml) to fully remove the $(4\text{-}MeC_6H_4)_3Bi$ which was also formed, then refiltered and rewashed with ether (50 ml), ethanol (2 x 20 ml), light petroleum (2 x 20 ml) and dried to yield (4-methylphenyl)bis(quinolin-8-olato)bismuth(III) (1.31 g, 78%). The $^1H$ nmr spectrum of the product taken a week later showed that the intensity of the resonances assigned to ethanol had decreased to give a mole ratio of 10 : 1 (Found: C, 49.9; H, 3.3; N, 4.7. $C_{25}H_{19}BiN_2O_2$ requires C, 51.0; H, 3.3; N, 4.8. $C_{25}H_{19}BiN_2O_2.(C_2H_6O)_{0.1}$ requires C, 51.0; H, 3.3; N, 4.7%). $\nu_{max}$ 1917w, 1624w, 1602m, 1585s, 1494s, 1423m, 1313s, 1269s, 1233s, 1186w, 1171m, 1135w, 1101vs, 1071w, 1061m, 1032m, 1016m, 982w, 962w, 898m, 823s, 802s, 789s, 751s, 725vs, 644m, 610w $cm^{-1}$. $^1H$ nmr (300 MHz) δ1.07, t, *J* 7.0 Hz, 3H, $CH_3$; 1.98, s, 3H, $CH_3$-Ar; 3.46, m, 2H, $CH_2$; 4.39, t, *J* 5.1 Hz, 1H, OH; 6.87, m, 4H, H5, 7; 7.17, d, *J* 7.6 Hz, 2H, H3', 5'; 7.41, t, *J* 7.9 Hz, 2H, H6; 7.60, dd, *J* 8.2, 4.5 Hz, 2H, H3; 8.33, d, *J* 8.3 Hz, 4H, H2', 6', 4; 9.28, d, *J* 3.7 Hz, 2H, H2. $^{13}C$ n.m.r. δ 20.86, $CH_3$-Ar; 110.29, C7; 116.07, C5; 121.23, C3; 129.70, C6; 131.27, C4a; 131.68, C3', 5'; 135.08, C2', 6'; 135.77, C4'; 137.62, C4; 142.36, C8a; 145.55, C2; 164.32, C8; 212.11, C1'. Mass spectrum *m/z* (no M), 444 (1%, $ArBi(Ox)^+$), 391 (2, $Ar_2Bi^+$), 360 (1), 300 (96, $ArBi^+$), 209 (100, $Bi^+$), 182 (22, $Ar_2^+$), 165 (10), 144 (10, $Ox^+$), 91 (10, $Ar^+$), 65 (7). UV/Vis (1.25 x $10^{-4}$ in $Me_2SO$) 323 (sh) (ε 3454), 339 (ε 4126), 391 nm) (ε 5925). (solid state) 400 nm. The solvent was removed from the filtrate to yield a yellow solid. This was extracted with water (6 x 10 ml) and ether (6 x 10 ml). NaCl was isolated from the aqueous

† Yields are calculated on the basis of reaction 1

‡ Prime numbers refer to atoms in the aryl group attached directly to bismuth extract (0.28 g, 90% by $AgNO_3$ titration). The ether washings were combined and evaporated to yield $(4\text{-}CH_3C_6H_4)_3Bi$ (1.22 g, 95%).

***$(4\text{-}MeC_6H_4)_2BiCl$ + Na(MeOx)***

A solution of Na(MeOx) (0.89 g, 4.9 mmol) with 15-crown-5 ether (2.16 g, 2.0 ml, 9.8 mmol) in ethanol (20 ml) was added to a suspension of $(4\text{-}MeC_6H_4)_2BiCl$ (2.10 g, 4.9 mmol) in ethanol (20 ml). The reaction mixture became a clear yellow colour before a yellow precipitate appeared. After stirring for 2 h, the reaction mixture was filtered and the resulting yellow solid was washed with ethanol (3 × 5 ml) and light petroleum (3 × 10 ml). The sample was further stirred in ether (50 ml) for 1 h to fully remove $(4\text{-}MeC_6H_4)_3Bi$. The suspension was filtered and the solid collected, rewashed with ether (2 × 20 ml), ethanol (2 × 10 ml) and light petroleum (2 × 20 ml) to yield (4-methylphenyl)bis(2-methylquinolin-8-olato)bismuth(III) (1.30 g, 86%) (Found: C, 52.6; H, 3.7; N, 4.5. $C_{27}H_{23}BiN_2O_2$ requires C, 52.6; H, 3.8; N, 4.5%). $\nu_{max}$ 1915w, 1738w, 1602w, 1586m, 1555s, 1503s, 1488w, 1429s, 1330s, 1297m, 1275s, 1210w, 1190m, 1170w, 1140w, 1103s, 1053m, 1014w, 974w, 961w, 919w, 866m, 832s, 796s, 758s, 749s, 738vs, 696w, 668w, 607m $cm^{-1}$. $^1H$ nmr (300 MHz) δ 2.01, s, 3H, $CH_3$-Ar; 2.83, s, 6H, $CH_3$; 6.90, d, *J* 7.7 Hz, 2H, H7; 7.01, d, *J* 7.9 Hz, 2H, H5; 7.24, d, *J* 7.6 Hz, 2H, H6; 7.40, t, *J*7.8 Hz, 2H, H3', 5'; 7.49, d, *J* 8.4 Hz, 2H, H3; 8.02, d, *J* 7.6 Hz, 2H, H2', 6', 8.34, d, *J* 8.4 Hz, 2H, H4. $^{13}C$ nmr δ 20.88, $CH_3$-Ar; 24.41, $CH_3$; 112.86, C7; 117.25, C5; 122.48, C3; 128.45, C6; 129.04, C4a; 132.17, C3', 5'; 134.67, C4'; 136.61, C2', 6'; 138.18, C4; 154.28, C2; 162.18, C8; C8a and C1' were not observed. Mass spectrum *m/z* (no M), 525 (1%, $Bi(MeOx)_2^+$), 458 (2, $ArBi(MeOx)^+$), 391 (1, $Ar_2Bi^+$), 367 (2, $Bi(MeOx)^+$), 336 (4, $BiI^+$), 300 (20, $ArBi^+$), 209 (30, $Bi^+$), 182 (22, $Ar_2^+$), 159 (100, $MeOxH^+$), 131 (63), 91 (20, $Ar^+$), 77 (10, $Ph^+$), 65 (18). U.v.-vis. ($8.04 \times 10^{-5}$ M in $Me_2SO$) 319 (sh) (ε 4214), 337 (sh) (ε 4649), 371 nm (ε 6054). (Solid state) 400 nm. The solvent was removed from the filtrate to yield a pale yellow solid. This was extracted with water (6 × 10 ml) and ether (6 × 10 ml). NaCl (0.29 g, 98% by $AgNO_3$ titration) was isolated from the aqueous washings. The solvent was removed from the ether extract to yield $(4\text{-}MeC_6H_4)_3Bi$ (1.13 g, 96%) identified by $^1H$ nmr spectroscopy.

***$(4\text{-}ClC_6H_4)_2BiCl$ + Na(Ox)***

A solution of Na(Ox) (0.50 g, 3.0 mmol) in ethanol (20 ml) with 15-crown-5 ether (1.32 g, 1.2 ml, 6.0 mmol) was added to a solution of $(4\text{-}ClC_6H_4)_2BiCl$ (1.72 g, 3.7 mmol) in ethanol (40 ml). A yellow precipitate gradually appeared and the reaction mixture was stirred for 2 h. The yellow solid was collected by filtration, washed with ethanol (3 × 5 ml), light petroleum (3 × 10 ml) and dried to yield an ethanol solvate of (4-chlorophenyl)bis(quinolin-8-olato)bismuth(III) (0.91 g, 93%) (Found: C, 44.1; H, 2.7; Cl, 5.4; N, 3.9. $C_{24}H_{16}BiClN_2O_2$ requires C, 47.4; H, 2.7; Cl, 5.8; N, 4.6. $C_{24}H_{16}BiClN_2O_2.C_2H_6O$ requires C, 47.7; H, 3.4; Cl, 5.4; N, 4.3%). $\nu_{max}$ 1914w, 1602m, 1566vs, 1494vs, 1420m, 1312vs, 1269vs, 1233s, 1171m, 1100vs, 1086s, 1050m, 1033w, 1008s, 960w, 888w, 884w, 823s, 804m, 788m, 749m, 725s, 644w $cm^{-1}$. $^1H$ nmr (300 MHz) δ 1.06, t, *J* 7.0 Hz, 3H, $CH_3$; 3.47, m, 2H, $CH_2$; 4.38, t, *J* 5.0 Hz, 1H, OH; 6.86, d, *J* 7.7 Hz, 2H, H7; 6.90, d, *J* 8.0 Hz, 2H, H5; 7.39, m,4H, H3', 5', 6; 7.60, dd, *J* 8.2, 4.5 Hz, 2H, H3; 8.33, d, *J* 8.0 Hz, 2H, H4; 8.47, d, *J* 8.0 Hz, 2H, H2', 6'; 9.30, d, *J* 3.9 Hz, 2H, H2. $^{13}C$ nmr δ 110.52, C7; 116.13, C5; 121.27, C3; 129.71, C6; 130.75, C3', 5'; 131.24, C4a, 131.35, C4'; 137.04, C2', 6'; 137.73, C4; 142.28, C8a; 145.71, C2; 164.10, C8; C1; not observed. Mass spectrum *m/z* (no M), 431 (2%, $Ar_2Bi^+$), 320 (60, $ArBi^+$), 222 (12, $Ar_2^+$), 209 (100, $Bi^+$), 152 (7), 111 (4, $Ar^+$), 76 (8); UV/Vis ($5.47 \times 10^{-5}$ M in $(CH_3)_2SO$) 325 (sh) (ε 4117), 339 (ε 4556), 389 nm (ε 6184). (Solid state) 390 nm.

***$(4\text{-}ClC_6H_4)_2BiCl$ + Na(MeOx)***

A solution of Na(MeOx) (0.36 g, 2.0 mmol) with 15-crown-5 ether (0.86 g, 0.8 ml, 3.9 mmol) in ethanol (25 ml) was added to a solution of $(4\text{-}ClC_6H_4)_2BiCl$ (0.92 g, 2.0 mmol) in ethanol (20 ml). A pale yellow precipitate gradually appeared and the reaction mixture was stirred for 2 h. The precipitate was collected by filtration, washed with ethanol (3 × 5 ml), light petroleum (3 × 10 ml) and dried to yield (4-chlorophenyl)bis(2-methylquinolin-8-olato)bismuth(III) (0.57 g, 91%) (Found: C, 49.0; H, 3.2; Cl, 5.7; N, 4.3. $C_{26}H_{20}BiClN_2O_2$ requires C, 49.0; H, 3.2; Cl, 5.6; N, 4.4%). $\nu_{max}$ 1587m, 1558s, 1502s, 1429s, 1328s, 1298m, 1274vs, 1238w, 1170w, 1104s, 1091s, 1053m, 1005m, 972w, 868w, 833s, 808m, 795w, 752s, 738s, 717m, 698w,

608m cm$^{-1}$. $^1$H nmr (300 MHz) δ 2.83, s, 6H, $CH_3$; 6.91, d, *J* 7.7 Hz, 2H, H7; 7.03, d, *J* 7.9 Hz, 2H, H5; 7.45, m, 6H, H3', 5', 3, 6; 8.17, d, *J* 7.9 Hz, 2H, H2', 6'; 8.32, d, *J* 8.4 Hz, 2H, H4. $^{13}$C nmr δ 24.46, $CH_3$; 113.15, C7; 117.40, C5; 122.54, C3; 128.49, C6; 129.07, C4a; 129.96, C4'; 131.33, C3', 5'; 136.70, C2', 6'; 138.29, C4; 141.71, C8a; 154.53, C2; 161.93, C8; 213.68, C1'. Mass spectrum *m/z* (no M), 525 (1%, $Bi(MeOx)_2^+$), 478 (2), 431 (<1, $Ar_2Bi^+$), 367 (2, $Bi(MeOx)^+$, 336 (1, $BiI^+$), 320 (11, $ArBi^+$), 222 (13, $Ar_2^+$), 209 (35, $Bi^+$), 159 (100, $MeOxH^+$), 131 (64), 112 (7, $ArH^+$), 103 (12), 77 (21, $Ph^+$), 51 (18). UV/Vis ($9.95 \times 10^{-5}$ M in $Me_2SO$) 319 (sh) (ε 6169), 336 (sh) (ε 6893), 370 nm (ε 8942). (Solid state) 388 nm. The solvent was removed from the filtrate to yield a pale yellow solid. This was extracted with water (6 × 10 ml) and ether (6 × 10 ml). The aqueous extract yielded NaCl (0.11 g, 96% by $AgNO_3$ titration) and the ether extract yielded $(4\text{-}ClC_6H_4)_3Bi$ (0.50 g, 94%) identified by $^1$H nmr spectroscopy.

### *In Vitro Growth Inhibition and In Vivo Antitumour Testing*

The methods for testing the compounds for growth inhibition against L1210 or the cisplatin – resistant derivative L1210/DDP mouse leukaemia cells *in vitro* and P388 mouse leukaemia *in vivo* have been described previously [15]. Briefly, for the cell culture testing, cells were exposed to the drug at several concentrations for 48 h, after which they were counted using a Coulter counter (Model ZM) and compared to control cells grown in the presence of only the vehicle. The IC50, or concentration causing 50% inhibition of cell growth, was determined from the curve of percentage growth versus drug concentration. The SKOV-3 human ovarian carcinoma cell line was maintained in α-minimum essential medium plus 15% foetal calf serum. For these growth inhibition studies, $5 \times 10^3$ exponentially growing cells in 100 μl medium were allowed to adhere in 96-well culture plates for 12 to 16 h at 37°C in a humidified incubator gassed with 10% CO2/ 90% air. Drugs were dissolved in $Me_2SO$ and diluted in medium to 10 concentrations over a 4-log range, and 100 μl of each drug solution was added to 5 wells. Cells were incubated for a further 72 h, after which viable cells were measured using the sulforhodamine B (SRB) assay [16] that measures cellular protein content. Briefly, cells were fixed with trichloroacetic acid and stained with SRB. Unbound dye was removed by washing with acetic acid, protein-bound dye was solubilised with Tris base, and the optical density was read at 550 nm using an automatic plate reader. The percentage cell growth inhibition was calculated as described above. All *in vitro* tests were done in duplicate, with repeats if greater than a 20% difference, and the results are reported as the mean.

All animal protocols were approved by the Institutional Animal Experimentation and Ethics Committee. For the animal studies with mouse leukaemia, DBA/2 mice received 106 P388 cells intraperitoneally (i.p.), and drugs were injected i.p. on days 1, 5, and 9. Although increased lifespan measured in days was the endpoint, animals were sacrificed prior to death, when movement was restricted by ascites. Controls received vehicle only, and %T/C was calculated as the ratio of survival time of treated animals over control animals, where compounds with %T/C greater that 125% were considered to have some activity.

For the *in vivo* antitumour activity against PC-6 plasmacytoma, female Balb/c mice (10-15 weeks old) were maintained in controlled atmospheric conditions and fed standard mouse chow and water *ad lib.*. The compounds were suspended by sonication in peanut oil. The murine PC6 plasmacytoma (obtained from L. Kelland, Institute of Cancer Research, Sutton, U K) was inoculated as 1 mm cubes subcutaneously on the flanks of the mice, and approximately 20 days later, mice with tumours were randomised into groups of 5 to 10 animals, which received either nothing (no-drug control), or an intraperitoneal injection of peanut oil at 10 ml/kg (vehicle control), cisplatin in saline at 6 mg/kg (positive control), or the test compounds at the maximum tolerated dose of 30 mg/kg. Eight to ten days later, mice were sacrificed and the tumours were dissected and weighed. The results were expressed as %T/C = mean tumour weight of the treated animals over mean tumour weight of the vehicle control group, where values less than 75% are considered to be worth further investigation.

### *Crystallography*

Intensity data for a yellow crystal (0.21 x 0.21 x 0.21 mm) were measured at room temperature on a Rigaku AFC6R diffractometer fitted with MoKα radiation (graphite monochromator, λ = 0.71073 Å) using the ω:2θ scan technique so that $\theta_{max}$ was 28.0°. No decomposition of the crystal occurred during the data collection and the data set was corrected for Lorentz and polarization effects [17], and for absorption employing an empirical procedure [18]. A total of 5905 data (5601 unique) were collected and of these, 3349 that satisfied the $I \geq 3.0\sigma(I)$ criterion were used in the subsequent analysis.

*Crystal data:* $C_{26}H_{21}BiN_2O_2$, $M$ = 602.4, monoclinic, space group $P2_1/c$, $a$ = 9.912(3) Å, $b$ = 15.004(2) Å, $c$ = 15.621(2) Å, $\beta$ = 106.92(1)°, $V$ = 2222.6(7) Å$^3$, $Z$ = 4, $D_{calc}$ = 1.800 g cm$^{-3}$, $F$(000) = 1160, $\mu$ = 79.39 cm$^{-1}$.
The structure was solved by direct-methods [19] and refined by a full-matrix least-squares procedure based on $F$ [17]. The non-hydrogen atoms were refined with anisotropic displacement parameters and hydrogen atoms were included in the model in their calculated positions (C-H 0.97 Å). The refinement was continued until convergence with sigma weights when $R$ = 0.032 and $R_w$ = 0.031. The maximum residual in the final difference map was 0.60 e Å$^{-3}$. Fractional atomic coordinates are listed in Table 1 and the numbering scheme employed is shown in Fig. 1 which was drawn with ORTEP [20] at 50 % probability ellipsoids. Data manipulation was performed with the teXsan program [17] installed on an Iris Indigo work station. Other crystallographic details, comprising fractional atomic coordinates for all atoms, thermal parameters, all bond distances and angles have been deposited at the Cambridge Crystallographic Data Centre; deposition no. 103306.

**TABLE 1.** Fractional atomic coordinates and $B_{eq}$ (Å$^2$)$^a$ values for [PhBi(MeOx)$_2$]

| Atom | x | y | z | $B_{eq}$ |
|---|---|---|---|---|
| Bi | 0.55978(3) | 0.33877(2) | 0.98078(2) | 2.718(5) |
| O(1) | 0.7362(5) | 0.2839(3) | 0.9397(3) | 3.7(1) |
| O(2) | 0.5333(5) | 0.4009(3) | 0.8489(3) | 3.7(1) |
| N(1) | 0.7033(6) | 0.2308(4) | 1.1005(3) | 2.9(1) |
| N(2) | 0.3287(6) | 0.4403(3) | 0.9307(4) | 3.1(1) |
| C(1) | 0.4170(7) | 0.2255(5) | 0.9194(5) | 3.2(2) |
| C(2) | 0.4267(9) | 0.1418(5) | 0.9585(6) | 4.6(2) |
| C(3) | 0.334(1) | 0.0746(5) | 0.9201(7) | 5.8(3) |
| C(4) | 0.232(1) | 0.0884(6) | 0.8418(7) | 6.2(3) |
| C(5) | 0.219(1) | 0.1706(7) | 0.8007(7) | 7.4(3) |
| C(6) | 0.314(1) | 0.2383(5) | 0.8403(6) | 5.3(2) |
| C(7) | 0.8414(7) | 0.2412(4) | 0.9971(5) | 3.1(2) |
| C(8) | 0.9660(8) | 0.2207(5) | 0.9799(5) | 4.2(2) |
| C(9) | 1.0762(9) | 0.1749(6) | 1.0412(6) | 5.4(2) |
| C(10) | 1.0626(9) | 0.1473(6) | 1.1206(6) | 5.3(2) |
| C(11) | 0.9382(8) | 0.1654(5) | 1.1447(5) | 3.9(2) |
| C(12) | 0.8264(7) | 0.2106(4) | 1.0817(4) | 3.0(2) |
| C(13) | 0.6863(8) | 0.2094(5) | 1.1783(5) | 3.6(2) |
| C(14) | 0.5495(9) | 0.2365(5) | 1.1948(5) | 4.7(2) |
| C(15) | 0.7923(10) | 0.1636(5) | 1.2442(5) | 4.6(2) |
| C(16) | 0.9149(10) | 0.1434(5) | 1.2266(5) | 4.7(2) |
| C(17) | 0.4306(8) | 0.4572(5) | 0.8108(5) | 3.6(2) |
| C(18) | 0.4243(9) | 0.4971(5) | 0.7293(5) | 4.7(2) |
| C(19) | 0.317(1) | 0.5573(6) | 0.6886(6) | 5.7(2) |
| C(20) | 0.2134(10) | 0.5802(5) | 0.7252(6) | 5.4(2) |
| C(21) | 0.2165(9) | 0.5428(5) | 0.8090(6) | 4.2(2) |
| C(22) | 0.3230(8) | 0.4799(5) | 0.8501(5) | 3.4(2) |
| C(23) | 0.2381(8) | 0.4625(5) | 0.9735(5) | 3.8(2) |
| C(24) | 0.2489(9) | 0.4187(6) | 1.0612(6) | 5.2(2) |
| C(25) | 0.1337(8) | 0.5268(6) | 0.9380(6) | 4.5(2) |
| C(26) | 0.1204(8) | 0.5652(5) | 0.8586(7) | 5.1(2) |

$a$ where $B_{eq} = 8\pi^2/3(U_{11}(aa^*)^2 + U_{22}(bb^*)^2 + U_{33}(cc^*)^2 + 2U_{12}aa^*bb^*\cos\gamma + 2U_{13}aa^*cc^*\cos\beta + 2U_{23}bb^*cc^*\cos\alpha)$

### Results and Discussion

### *Preparation and characterisation of arylbismuth oxinates*

A range of new arylbismuth(III) oxinates, ArBi$(Ox)_2$ (Ar = *p*-Me$C_6H_4$ or *p*-$Cl_6H_4$) and ArBi$(MeOx)_2$ (Ar = Ph, *p*-$Me_6H_4$, or *p*-Cl$C_6H_4$) have been prepared by reaction of diarylbismuth(III) chlorides with Na(Ox) or Na(MeOx) in ethanol in the presence of 15-crown-5.

$$2Ar_2BiCl + 2Na(Ox\ or\ MeOx) \rightarrow ArBi(Ox\ or\ MeOx)_2 + Ar_3Bi + 2NaCl \qquad (1)$$

The crown ether prevented retention of sodium chloride by the arylbismuth oxinate [1]. In representative cases, the amount of sodium chloride produced was shown to be *ca.* 90% as was the yield of $Ar_3Bi$. Reaction (1) rather than reaction of Ar$BiCl_2$ with Na(Ox or MeOx) was chosen to avoid formation of Bi$(Ox)_3$ or Bi$(MeOx)_3$ [1], which cannot be separated from ArBi(Ox or MeOx$)_2$ owing to their mutual low solubilities. The $Ar_3Bi$ compounds can be recycled by reaction with $BiCl_3$ to give the reactants, $Ar_2BiCl$. Most complexes were analytically pure except RBi$(Ox)_2$ (R = *p*-Me$C_6H_4$ or *p*-Cl$C_6H_4$) where use of the crown ether makes it unlikely that the low carbon analyses were due to sodium halide retention. Satisfactory $^1H$ and $^{13}C$ NMR spectra and UV/Vis spectra (showing chelation of Ox and MeOx) [21,22] were obtained for all complexes including RBi$(Ox)_2$ (R = *p*-Me$C_6H_4$ or *p*-Cl$C_6H_4$). A COSY $^1H$ NMR spectrum of PhBi$(MeOx)_2$ showed that H2,6 of the phenyl group was at lower frequency than H4 of MeOx by contrast with the reported spectrum [1] of PhBi$(Ox)_2$.

### *Molecular structure of PhBi(MeOx)$_2$*

The molecular structure of PhBi$(MeOx)_2$ is illustrated in Figure 1 and selected interatomic parameters are listed in Table 2. This is the first structurally characterised bismuth(III) oxine complex. Only the bismuth(V) derivatives, $Ph_3$Bi(Ox or MeOx)Cl have been analysed crystallographically previously [21,23].

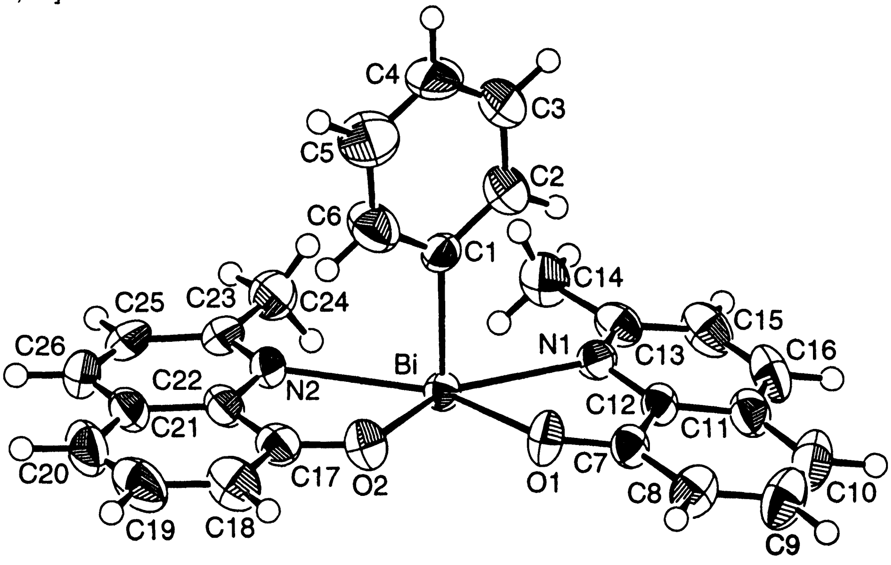

**Figure 1.** The molecular structure of PhBi$(MeOx)_2$

The bismuth atom is five coordinate, with distorted square pyramidal stereochemistry. The square plane is defined by a $N_2O_2$ donor set derived from two chelating MeOx anions, and the bismuth atom lies 0.0565(2) Å above the least-squares plane through the $N_2O_2$ atoms (mean deviation: 0.014 Å). The square plane is significantly distorted towards a trapezoidal geometry owing to the presence of disparate Bi—O and Bi—N distances. This disparity is manifested in the wide N—Bi—N angle of 145.0(2)° and may arise in order to minimise intramolecular repulsions between the methyl groups. The phenyl group is almost symmetrically inclined with respect to the $BiN_2O_2$ plane, forming a dihedral angle of 95.6° with it.

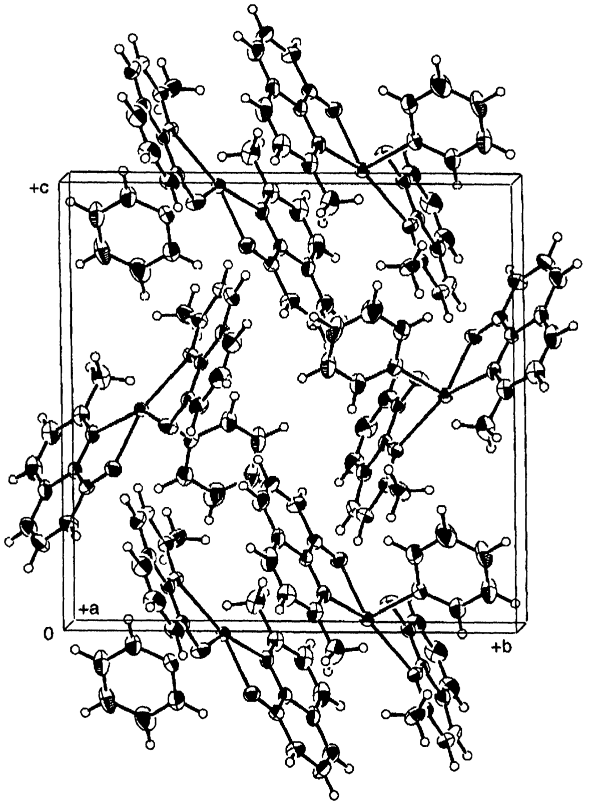

**Figure 2.** Unit cell contents for $PhBi(MeOx)_2$

**TABLE 2.** Selected interatomic parameters (Å, deg.) for $PhBi(MeOx)_2$

| | | | |
|---|---|---|---|
| Bi-O(1) | 2.191(4) | Bi-O(2) | 2.207(4) |
| Bi-N(1) | 2.569(5) | Bi-N(2) | 2.671(5) |
| Bi—C(1) | 2.241(7) | O(1)—C(7) | 1.325(8) |
| O(2)—C(17) | 1.322(8) | N(1)—C(12) | 1.369(8) |
| N(1)—C(13) | 1.315(8) | N(2)—C(22) | 1.378(8) |
| N(2)—C(23) | 1.310(9) | | |
| O(1)—Bi—O(2) | 76.9(2) | O(1)—Bi—N(1) | 69.9(2) |
| O(1)—Bi—N(2) | 144.9(2) | O(1)—Bi—C(1) | 92.7(2) |
| O(2)—Bi—N(1) | 146.8(2) | O(2)—Bi—N(2) | 68.1(2) |
| O(2)—Bi—C(1) | 91.8(2) | N(1)—Bi—N(2) | 145.0(2) |
| N(1)—Bi—C(1) | 88.9(2) | N(2)—Bi—C(1) | 85.8(2) |
| Bi—O(1)—C(7) | 121.4(4) | Bi—O(2)—C(17) | 123.8(4) |
| Bi—N(1)—C(12) | 108.9(4) | Bi—N(1)—C(13) | 129.4(5) |
| C(12)—N(1)—C(13) | 120.4(6) | Bi—N(2)—C(22) | 108.5(4) |
| Bi—N(2)—C(23) | 130.7(5) | C(22)—N(2)—C(23) | 120.3(6) |

A stereochemically active lone pair of electrons, localised on the bismuth centre, may be expected to occupy a position opposite the phenyl substituent. The overall geometry found for $PhBi(MeOx)_2$ is similar to that in the closely related compounds *bis*(1-oxopyridine-2-thiolato)phenylbismuth(III) [24] and chlorobis(2-phenylquinolin-8-thiolato)bismuth(III) [25]. Further, the association between centrosymmetrically related molecules is similar to that in bis(1-oxopyridine-2-thiolato)phenylbismuth(III). Thus, in the lattice of $PhBi(MeOx)_2$ centrosymmetrically related molecules approach each other so as to bring the two planar portions of the molecules into close proximity. The average separation between the two $Bi(MeOx)_2$ moieties is 3.20 Å, which is smaller than the sum of two phenyl ring van der Waals radii [26], suggesting significant $\pi$-$\pi$ interactions between them.

The bismuth-carbon distance is comparable with that of the related bis(1-oxopyridine-2-thiolato)phenylbismuth(III) and the average bond distance [27] of $Ph_3Bi$ (2.26 Å). Perhaps surprisingly the Bi-O distances are comparable with those of $Ph_3Bi$(Ox or MeOx)Cl (2.175(7) [23] and 2.19(2) [21], hence the effects of both a lower oxidation state and lower coordination number in $PhBi(MeOx)_2$ appear to cancel each other. However <Bi-N> is significantly shorter than Bi-N of $Ph_3Bi$(Ox or MeOX)Cl (2.807(10) [23] and 2.71(2) [21]) suggesting a stronger interaction in the bismuth(III) compound. Nevertheless <Bi-N> is rather larger than Bi-N (2.533(6)Å) in 2-(2'-pyridyl)phenylbismuth(III) bis(N,N-diethyldithiocarbamate) [28] despite the lower coordination number in the present compound. Possibly the steric effect of the methyl groups causes some lengthening.

### Growth Inhibition and Antitumour Testing

### *In vitro examination*

The results of testing several (quinolin-8-olato)bismuth(III) compounds against L1210 mouse leukaemia cells, the corresponding cisplatin resistant line L1210/DDP, and the human ovarian carcinoma (SKOV-3) are summarised in Table 3.

**TABLE 3.** *In vitro* growth inhibition results for bismuth(III) compounds against L1210, L1210/DDP and SKOV-3

| Compound[a] | | $IC_{50}/\mu M^b$ | |
|---|---|---|---|
| | L1210 | L1210/DDP | SKOV-3 |
| H(Ox) | 4.1 | 0.77 | 6.8 |
| H(MeOx) | 8.3 | 13 | 85 |
| $Bi(Ox)_3$ | 0.26 | 0.33 | 0.29 |
| $Bi(Ox)_2I$ | 0.25 | 0.22 | 0.77 |
| $PhBi(Ox)_2$.EtOH | 0.19 | 0.28 | 0.75 |
| $PhBi(MeOx)_2$ | 0.56 | 0.37 | 2.9 |
| $[NaPhBi(Ox)_3]$ | 0.34 | 0.12 | 0.64 |
| $[KPhBi(Ox)_3]$ | 0.35 | 0.27 | 0.66 |
| $Bi(OH)_3$ | > 5 | > 5 | n.d. |
| $Ph_3Bi$ | 42 | n.d. | n.d. |
| $Ph_2BiI$ | 0.29 | 0.31 | n.d. |
| $PhBiI_2$ | 1.3 | 1.1 | n.d. |
| cisplatin | 0.6 | 6.7 | 3.1 |

[a] Compounds administered as solutions in < 1% $Me_2SO$ in media.
[b] $IC_{50}$ = minimum concentration (μM) required to inhibit the growth of cancer cells by 50%.
n.d no data

Most of the compounds showed excellent activity when compared with the clinically used cisplatin. H(Ox) shows very good activity against L1210/DDP but H(MeOx) is much less effective especially against L1210/DDP and SKOV-3, perhaps owing to steric repulsion by the 2-methyl substituent. Coordination to bismuth enhances the activity of the free H(Ox) and H(MeOx) ligands. Similarly, the microbial or fungicidal activity of H(Ox) is enhanced by co-administrations of metals [29]. Enhancement of the *in vivo* anticancer activity of 6-mercaptopurine by bismuth(III) is known [4]. The possibility that bismuth is enhancing delivery of active H(Ox) or H(MeOx), as proposed for 6-mercaptopurine [4], can be ruled out in view of the very poor activity of H(MeOx) towards DDP and SKOV-3 when compared with the high activities of $PhBi(MeOx)_2$. This is reinforced by the high activity observed for $Ph_2BiI$ and $PhBiI_2$. The dependence of activity on the ligands varies as

$Bi(OH)_3 \sim Ph_3Bi < PhBiI_2 < PhBi(MeOx)_2 < Ph_2BiI \sim PhBi(Ox)_2$

Thus, the activity of monophenylbismuth(III) compounds, $PhBiX_2$, is greater for X = Ox or MeOx than for X=I. It is possible that a partially hydrolysed 'in situ-delivered' mono- or diaryl-bismuth(III) compound is the active species, reinforced in the case of PhBi(Ox or $MeOx)_2$ by the activity of H(Ox or MeOx). Interestingly the bimetallic complexes [1] $[MPhBi(Ox)_3]$ (M = Na or K) have comparable activities to $PhBi(Ox)_2$, suggesting that their activity may involve similar species. Perhaps triphenylbismuth is inactive because it is inert to hydrolysis.

### *In vivo activity*

The results of testing $PhBi(Ox)_2{\cdot}EtOH$ and $[KPhBi(Ox)_3]$ against the solid mouse plasmacytoma, ADJ/PC6 are given in Table 4.

**TABLE 4.** *In vivo* testing results for bismuth(III) compounds, administered as a suspension in peanut oil, against PC6 in mice

| Compound | Dose | %T/C[a] |
|---|---|---|
| $PhBi(Ox)_2.EtOH$ | 30 mg/kg i.p. | 89 |
| $[KPhBi(Ox)_3]$ | 30 mg/kg i.p. | 146 |
| cisplatin | 6 mg/kg i.p. | 0.5 |

[a] %T/C = mean tumour weight of drug treated animal/mean tumour weight of controls, where values less than 75% are considered to indicate activity

Disappointingly, the compounds are inactive. This may be attributable to delivery problems as the complexes were completely insoluble in the delivery medium. The bimetallic $[KPhBi(Ox)_3]$ was also tested against P388 leukaemia (Table 5), with the compound delivered intraperitoneally as a solution in $Me_2SO$. Three separate concentrations were examined including the maximum tolerated dose but no activity was detected. These results contrast the reported *in vivo* activity of 6-mercaptopurinebismuth(III) complexes [4], the methylbismuth(III) thiolates [7], and, more closely relevant, $Ph_2BiO_2CR$ (R = Et, iPr or Ph) [6], for which activity against P388 leukaemia (T/C 147-168% for doses of 50-200 mg/kg) was recorded [6]. Of relevance to the latter observation is that diphenylbismuth(III) carboxylates have been shown to be highly sensitive to hydrolytic cleavage of aryl groups [30]. For PhBi(Ox or $MeOx)_2$, the contrast between the excellent activity *in vitro* and the lack of activity *in vivo* is dramatic, and points to delivery problems in animal models. This has not been overcome by dissolution of the low solubility drugs in $Me_2SO$, where precipitation on injection into aqueous biological fluids may deactivate the compounds.

**TABLE 5.** *In vivo* testing results for $[KPhBi(Ox)_3]$, dissolved in dimethyl sulfoxide, against P388 leukaemia in mice

| Dose | %T/C[a] |
|---|---|
| 20 mg/kg i.p. | 105 |
| 10 mg/kg i.p. | 111 |
| 2 mg/kg i.p. | 108 |
| 5-Fluorouracil[b] | 200 |

a %T/C was calculated as the ratio of survival time of treated animals over control animals, where compounds with %T/C greater that 125% are considered to have some activity
b positive control

**Acknowledgements**

The Australian Research Council is thanked for support of the crystallographic facility.

**References**

1. K.A. Smith, G.B. Deacon, W.R. Jackson and J.M. Miller, *Aust. J. Chem.*, 1996, **49**, 231.
2. I. Haiduc and C. Silvestru, *Organometallics in Cancer Chemotherapy*, Vol. 1, Cht., pt3, Ch. 9, pp. 243-244 (CRC Press, Ind.: Florida, 1989); U. Wormser and I. Nir, *Pharmacology and Toxicology of Organic Bismuth, Arsenic, and Antimony Compounds*, in *The Chemistry of Organic Arsenic, Antimony, and Bismuth Compounds*, Ed. S. Patai, Wiley, NY, 1994, Ch. 18 and references therein.
3. T. Klapötke, *Biol Met.*, 1988, **1**, 69 and references therein.
4. S. Kirschner, Y.-K. Wei, D. Francis and J.G. Bergman, *J. Med. Chem.*, 1966, **9** 369.
5. F. Huber, G. Roge, L. Carl, G. Atassi, F. Spreafico, S. Filippeschi, R. Barbieri, A. Silvestri, E. Rivarola, G. Ruisi, F. Di Bianca and G. Alonzo, *J. Chem. Soc., Dalton Trans.,* 1985, 523.
6. F. Huber, unpublished data (personal communications to G.B. Deacon).
7. P. Köpf-Maier and T. Klapötke, *Inorg. Chim. Acta*, 1988, **152**, 49.
8. M. Yamato, J. Ando, K. Sakaki, K. Hashigaki, Y. Wataya, S. Tsukagoshi, T. Tashiro and T. Tsuruo, *J. Med. Chem.*, 1992, **35**, 267.
9. J. Nordenberg, A. Novogrodsky, E. Beery, M. Patia, L. Wassserman and A. Warshawsky, *Eur. J. Cancer* 1990, **26**, 905.
10. G.F. Baxter, *Chemistry in Britain*, 1992, 445; J. Alper, *Science*, 1993, **260**, 159, G. Vines, *New Scientist*, 1994, 12.
11. G.M. Bouvier, C. Berin, A. Beugnet, J. Cordier and F. Derrieunic, *The toxicity of bismuth: a survey conducted by INSERM*, in *Science et Vie*, July 1976.
12. L.D. Freedman and G.O. Doack, *Chem. Rev.*, 1982, **82**, 15.
13. F. Challenger and C.F. Allpress, *J. Chem. Soc.*, 1915, **107**, 16.
14. A.O. Aliprantis and J.W. Canary, *J. Am. Chem. Soc.*, 1994, **116**, 6985.
15. L.K. Webster, G.B. Deacon, D.P. Buxton, B.L. Hillcoat, A.M. James, I.A.G. Roos, R.J. Thomson, L. P.G. Wakelin and T. L. Williams, *J Med Chem.*, 1992, **35**, 3349
16. P. Skehan, R. Storeng, D. Scudiero, A. Monks, J. McMahon, D. Vistica, J.T. Warren, H. Bokesch, S. Kenney, and M.R. Boyd, *J. Natl. Cancer Inst.*, 1990, **82**, 1107.
17. *teXsan, Single Crystal structure analysis software*, Version 1.6 (1993), Molecular Structure Corporation, The Woodlands, TX, USA.
18. N. Walker and D. Stuart, *Acta Crystallogr.*, 1983, *A***39**,158.
19. M.C. Burla, M. Camalli, G. Cascarano, C. Giacovazzo, G. Polidori, R. Spagna and D. Viterbo, *J. Appl. Cryst.*, 1989, **22**, 389.
20. C.K. Johnson, ORTEP. Report ORNL-5138 (1976), Oak Ridge National Laboratory, TN, USA.
21. G. Faraglia, R. Graziani, L. Volponi and U. Casellato, *J. Organomet. Chem.*, 1983, **253**, 317.
22. T. Moeller and A.J. Cohen, *J. Am.Chem. Soc.*, 1950, **72**, 3546.
23. D.H.R. Barton, B. Charpiot, E.T.H. Dau, W.B. Motherwell, C. Pascard and C. Pinchon, *Helv. Chim. Acta*, 1984, **67**, 586.
24. J.D. Curry and R.J. Jandacek, *J. Chem. Soc. Dalton Trans.*, 1972, 1120.
25. I. Berzina, O.G. Matyukhina, V. Bel'skii, J. Asaks and Yu.A. Bankovskii, Latv. PSR Zinat. Akad. Vestis, Kim. Ser., 1985, **2**, 161; *Chem. Abstr.*, 1985, **103**, 31565n.
26. L. Pauling. *The nature of the chemical bond*, 3rd Ed., Ithaca, NY, 1960.
27. P.G. Jones, A. Blaschette, D. Henschel and A. Weitze, *Z. Krist.*, 1995, **210**, 377.
28. M. Ali, W.R. McWhinnie, A.A. West and T.A. Hamor, *J. Chem. Soc. Dalton Trans.*, 1990, 899.
29. A. Albert, *J. Med. Chem.*, 1982, **25**,
30. G.B. Deacon, W.R.Jackson and J.M. Pfeiffer, *Aust. J. Chem.*, 1984, **37**, 527.

**Received: August 28, 1998 - Accepted: September 30, 1998 - Received in revised camera-ready format: October 6, 1998**

